# Marked improvement of oral intake with nivolumab monotherapy in a patient with microsatellite instability‐high gastric cancer with insufficient oral intake

**DOI:** 10.1002/ccr3.3399

**Published:** 2020-11-18

**Authors:** Takatsugu Ogata, Yukiya Narita, Kazunari Misawa, Waki Hosoda, Kei Muro

**Affiliations:** ^1^ Department of Clinical Oncology Aichi Cancer Center Hospital Aichi Japan; ^2^ Department of Surgery Aichi Cancer Center Hospital Aichi Japan; ^3^ Department of Pathology and Molecular Diagnostics Aichi Cancer Center Hospital Aichi Japan

**Keywords:** advanced gastric cancer, high microsatellite instability, immune checkpoint inhibitors, insufficient oral intake

## Abstract

Although immune checkpoint inhibitors are commonly less effective for patients with a poor general condition, they can be effective and should be considered for poor general conditions in the case of MSI‐H tumor.

## Graphical abstract

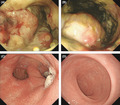

## 1 INTRODUCTION

We report herein the case of marked improvement of oral intake via third‐line nivolumab monotherapy in a gastric cancer patient with insufficient oral intake and European Cooperative Oncology Group performance status 2. We should consider immune checkpoint inhibitors for microsatellite instability‐high gastric cancer, regardless of the patient's general condition.

Gastric cancer has one of the worst prognoses among all cancer types. Gastrointestinal obstruction due to primary obstruction or peritoneal metastasis is a common complication of advanced gastric cancer. Patients with gastrointestinal obstruction often experience insufficient oral intake, and these patients tend to have worse prognosis. In the JCOG0106 study,^1^ sufficient or insufficient oral intake was defined based on whether drip infusion for nutrition support was performed, and the improvement of oral intake was defined as drip infusion not being indicated for > 7 days in patients who previously had insufficient oral intake. Some studies have shown improvement of oral intake with systemic chemotherapy, but these studies were in patients receiving first‐ or second‐line treatment.^1^, ^2^, ^3^, ^4^, ^5^, ^6^, ^7^ There have been no published cases of improvement of oral intake with third‐ or later‐line chemotherapy.

Immune checkpoint inhibitors (ICIs) have become widely used for the treatment of various cancers. Nivolumab monotherapy for heavily pretreated gastric cancer was approved as a third‐ or later‐line treatment in Japan in September 2017. However, according to the ATTRACTION‐2 study, the response was very limited.^8^ Further, although there are some reports of good response to ICIs in gastric cancer patients, there is no report on the improvement of oral intake by nivolumab monotherapy.^9^

To our best knowledge, the current case is the first report of marked improvement of oral intake by third‐line nivolumab monotherapy in a gastric cancer patient with insufficient oral intake.

## 2 CASE HISTORY

A 76‐year‐old woman presented to our hospital with a stomachache and loss of appetite for 3 months. She was admitted with insufficient oral intake for careful examination and treatment. She had rheumatoid arthritis treated with methotrexate.

### 2.1 Differential diagnosis, investigations, and treatment

**Figure 1:**
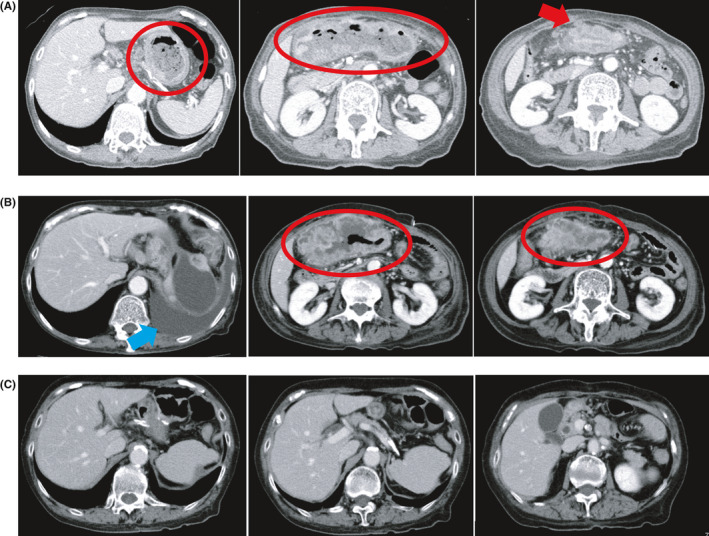
Computed tomography results. (A) At diagnosis: gastric wall thickening (red circle), invasion of the abdominal wall, and regional lymphadenopathy (red arrow). (B) Before nivolumab monotherapy: gastric wall thickening (red circle), invasion of the abdominal wall, regional lymphadenopathy, and pleural effusion (blue arrow). (C) Two years after nivolumab monotherapy: complete response

**Figure 2:**
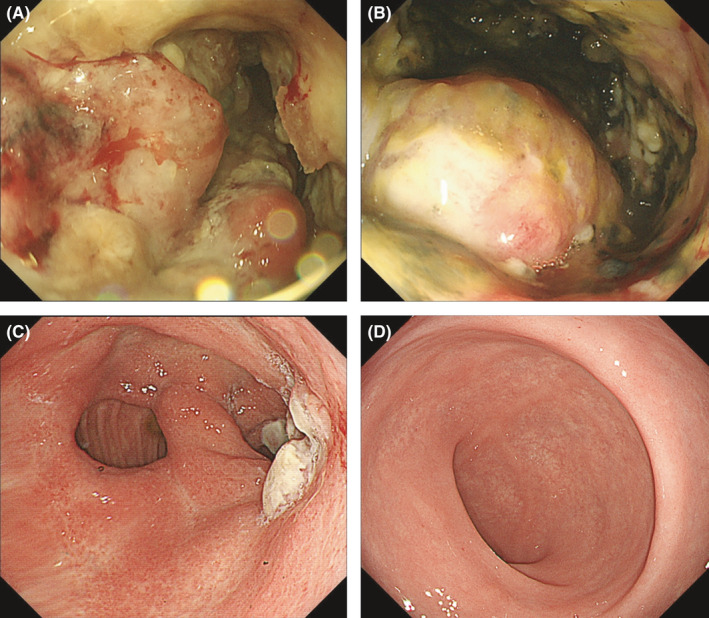
Upper gastrointestinal endoscopy results. (A) At diagnosis: Bormann type 3 gastric cancer was observed. (B) Before nivolumab monotherapy: disease progression. (C) Six months after nivolumab monotherapy: tumor shrinkage. (D) Two years after nivolumab monotherapy: complete response (endoscopic biopsy revealed no residual tumor cells)

**Figure 3:**
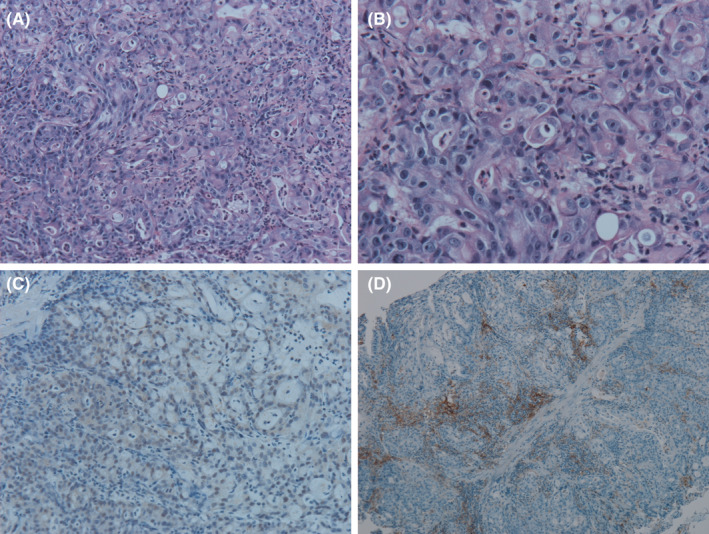
Histology and immunohistochemistry of the gastric mucosal biopsy specimen. (A) Hematoxylin‐eosin stain (low‐power field) indicating poorly differentiated adenocarcinoma. (B) Hematoxylin‐eosin stain (high‐power field) indicating poorly differentiated adenocarcinoma. (C) Epstein‐Barr virus‐encoded RNA in situ hybridization was negative. (D) Programmed death‐ligand 1 staining; the combined positive score ≥ 10

Physical examination revealed no significant findings. The laboratory data on admission are summarized in Table 1. Computed tomography (CT) revealed gastric wall thickening, invasion of the abdominal wall, and regional lymphadenopathy (Figure 1A). Gastrointestinal endoscopy (GIE) revealed Bormann type 3 gastric cancer (Figure 2A). Biopsy of the gastric mucosa showed poorly differentiated adenocarcinoma (Figure 3A, 3B). Immunohistochemical analysis (HercepTest, Dako, Glostrup, Denmark) showed no expression of human epidermal growth factor receptor 2 (score = 0), and *HER2/neu* amplification was confirmed negative using dual color in situ hybridization (INFORM HER2 Dual ISH DNA Probe Cocktail Assay).^10^ The Epstein‐Barr encoding region in situ hybridization was also negative (Figure 3C).^11^ Programmed death‐ligand 1 (PD‐L1) protein expression on adenocarcinoma cells was assessed using PD‐L1 IHC 22C3 pharmDx (Agilent Technologies; Carpinteria, CA, USA), and the combined positive score (CPS) was 10 (Figure 3D).^12^ Microsatellite instability was high (MSI‐IVD Kit, FALCO^Ⓡ^). Therefore, she was diagnosed with unresectable advanced microsatellite instability‐high (MSI‐H) gastric cancer.^12^

**Table 1:** Laboratory data on admission

**Hematology**		**Normal range**
White blood cells	8570/μL	3300‐8600/μL
Neutrophils	82.3%	
Eosinophils	0.5%	
Basophils	0.2%	
Monophils	5.3%	
Lymphocytes	11.7%	
Red blood cells	321 × 10^4^/μL	386‐492 × 10^4^/μL
Hemoglobin	8.7 g/dL	11.6‐14.8 g/dL
Platelets	30.2 × 10^4^/μL	15.8‐34.8 × 10^4^/μL
**Coagulation**		**Normal range**
PT	91.6%	80.0%‐120.0%
APTT	28.8 sec	25.0‐38.0 sec
Fibrinogen	517.6 mg/dL	200.0‐400.0 mg/dL
**Biochemistry**		**Normal range**
Total protein	5.1 g/dL	6.7‐8.3 g/dL
Albumin	2.3 g/dL	4.0‐5.0 g/dL
Total bilirubin	0.3 mg/dL	0.3‐1.2 mg/dL
AST	15 U/L	13‐33 U/L
ALT	6 U/L	6‐27 U/L
LDH	217 U/L	119‐229 U/L
BUN	13 mg/dL	8‐22 mg/dL
Creatinine	0.46 mg/dL	0.40‐0.70 mg/dL
Na	139 mmol/L	138‐146 mmol/L
K	4.0 mmol/L	3.6‐4.9 mmol/L
Ca	8.0 mg/dL	8.7‐10.3 mg/dL
CRP	3.71 mg/dL	<0.30 mg/dL
**Tumor markers**		**Normal range**
CEA	2.3 ng/mL	<5.0 ng/mL
CA19‐9	42.2 U/mL	<37.0 U/mL
CA125	61.0 U/mL	<26.9 U/mL

Abbreviations: ALT, alanine aminotransferase; APTT, activated partial thromboplastin time; AST, aspartate aminotransferase; BUN, blood urea nitrogen; Ca, calcium; CA125, cancer antigen 125; CA19‐9, carbohydrate antigen 19‐9; CEA, carcinoembryonic antigen; CRP, C‐reactive protein; K, potassium; LDH, lactate dehydrogenase; Na, sodium; PT, prothrombin time.

She underwent gastrojejunal bypass surgery in July 2017, but her oral intake did not sufficiently improve. Therefore, total parenteral nutrition (TPN) was started. A month after the bypass, mFOLFOX6 therapy (oxaliplatin 85 mg/m^2^ intravenous [IV], *l*‐leucovorin 200 mg/m^2^ IV, and 5‐fluorouracil 400 mg/m^2^ IV bolus followed by 2400 mg/m^2^ over 46 hours starting on Day 1, every 2 weeks) was started as first‐line treatment. However, CT revealed disease progression after six cycles. Paclitaxel therapy (paclitaxel 80 mg/m^2^ on days 1, 8, and 15 every month) as second‐line treatment was also ineffective at the first evaluation after 3 cycles. Her general condition did not improve, and she needed TPN after progression on paclitaxel therapy. Although her Eastern Cooperative Oncology Group (ECOG) performance status (PS) was 2, nivolumab monotherapy (3 mg/m^2^ every 2 weeks until October 2018 and 240 mg/body every 2 weeks from November 2018) was administered as third‐line treatment. Laboratory tests, CT, and GIE before the first administration of nivolumab are summarized in Table 2, Figure 1B, and Figure 2B.

**Table 2:** Laboratory data at the first administration of nivolumab

**Hematology**		**Normal range**
White blood cells	17020/μL	3300‐8600/μL
Neutrophils	92.0%	
Monophils	3.5%	
Lymphocytes	4.5%	
Red blood cells	253 × 10^4^/μL	386‐492 × 10^4^/μL
Hemoglobin	7.0 g/dL	11.6‐14.8 g/dL
Platelets	31.7 × 10^4^/μL	15.8‐34.8 × 10^4^/μL
**Tumor markers**		**Normal range**
CEA	6.9 ng/mL	<5.0 ng/mL
CA19‐9	52.0 U/mL	<37.0 U/mL
**Biochemistry**		**Normal range**
Total protein	4.0 g/dL	6.7‐8.3 g/dL
Albumin	1.4 g/dL	4.0‐5.0 g/dL
Total bilirubin	0.2 mg/dL	0.3‐1.2 mg/dL
AST	31 U/L	13‐33 U/L
ALT	24 U/L	6‐27 U/L
LDH	192 U/L	119‐229 U/L
BUN	13 mg/dL	8‐22 mg/dL
Creatinine	0.41 mg/dL	0.40‐0.70 mg/dL
Na	135 mmol/L	138‐146 mmol/L
K	4.0 mmol/L	3.6‐4.9 mmol/L
Ca	7.4 mg/dL	8.7‐10.3 mg/dL
		

Abbreviations: ALT, alanine aminotransferase; AST, aspartate aminotransferase; BUN, blood urea nitrogen; Ca, calcium; CA19‐9, carbohydrate antigen 19‐9; CEA, carcinoembryonic antigen; K, potassium; LDH, lactate dehydrogenase; Na, sodium.

### 2.2 Outcome and follow‐up

**Figure 4:**
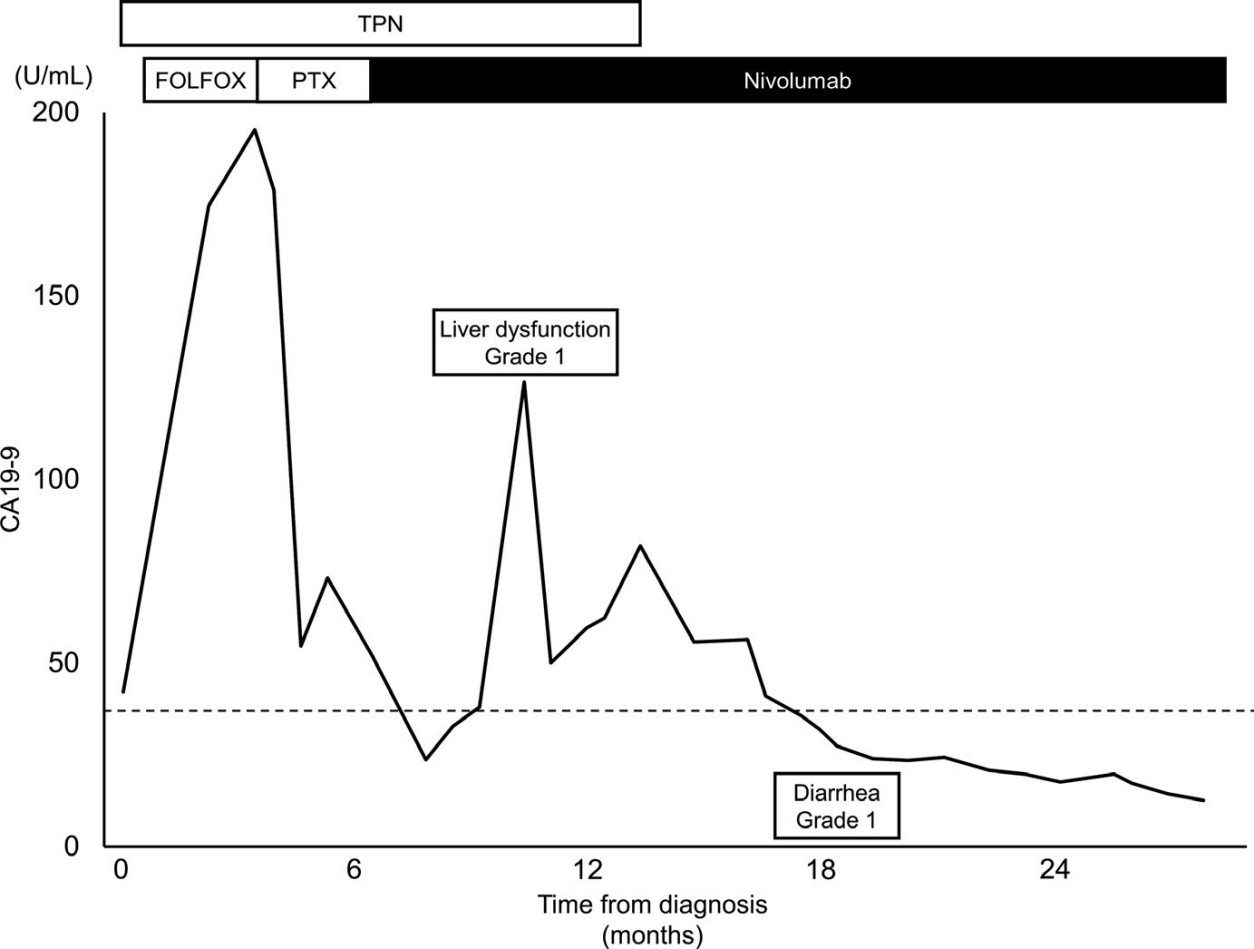
Changes in CA19‐9 level during the patient's course of treatment. CA19‐9, carbohydrate antigen 19‐9; TPN, total parenteral nutrition; PTX, paclitaxel

After four cycles of nivolumab, CT showed that the tumor had shrunk significantly. Her ECOG PS improved from 2 to 0. After 6 months, her oral intake problem had completely improved, and TPN was no longer needed. Upper gastrointestinal endoscopy indicated tumor shrinkage (Figure 2C). CT and upper gastrointestinal endoscopy revealed no residual tumor 2 years after the surgery (Figures 1C and 2D). These findings support that nivolumab monotherapy caused a complete response. She is currently receiving nivolumab monotherapy and has sufficient oral intake (Figure 4).

Facial paralysis occurred 7 months after nivolumab administration, and we diagnosed it as Bell's paralysis not related to nivolumab. She developed grade 1 liver dysfunction and grade 1 diarrhea as immune‐related adverse events (irAEs).

## 3 DISCUSSION

We report herein a case of marked improvement of oral intake by third‐line nivolumab monotherapy in a patient with MSI‐H gastric cancer with insufficient oral intake. The first‐ and second‐line treatments were not effective, but she achieved a complete improvement of oral intake with nivolumab and had a complete tumor response after nivolumab monotherapy. To our best knowledge, this is the first case report on improved oral intake from third‐line nivolumab therapy.

Insufficient oral intake is one of the most common complications of gastric cancer. The main causes of insufficient oral intake are gastric outlet obstruction and peritoneal metastasis. Insufficient oral intake is a poor prognostic factor, and Shitara et al reported that the median overall survival (OS) was significantly shorter in patients with insufficient oral intake than in those with sufficient oral intake (5.0 months vs. 12.7 months, *P* < .05).^3^ In previous studies, the rate of improvement of oral intake with first‐line treatment was 32%‐85% (Table 3).^1^, ^2^, ^3^, ^4^, ^5^, ^6^, ^7^ Platinum‐containing regimens seem to be more effective than platinum‐free regimens for the improvement of oral intake (Table 3). However, there are no reports of improvement of oral intake in patients receiving third‐ or later‐line treatment. In this case, oral intake was not improved by first‐ and second‐line treatment; additionally, the patient experienced disease progression. However, oral intake was completely improved with third‐line nivolumab monotherapy, and complete tumor response was achieved as observed on imaging studies.

**Table 3:** Improvement rate of oral intake with first‐line systemic chemotherapy

Trials	Phase	Regimen	Improvement rate	Reference
JCOG0106	III	5‐FU ci	41%	1
		5‐FU + MTX	57%	
JCOG1108/ WJOG7312G	II/III	5‐FU + LV	37%	2
		FLTAX	32%	
Arai et al	retro	5‐FU	43%	3
		5‐FU + platinum	64%	
Shitara et al	retro	Any	40%	4
Yukami et al	retro	FOLFOX	72%	5
Osumi et al	retro	mFOLFOX6	85%	6
Iwasa et al	retro	Any	33%	7

Note

5‐FU ci, 800 mg/m^2^/day on days 1‐5, every 4 weeks; MTX + 5‐FU, methotrexate 100 mg/m^2^ and 5‐FU 600 mg/m^2^, every week; Best available 5‐FU, 5‐FU ci, or MTX + 5‐FU; PTX, 80 mg/m^2^/day on days 1, 8, and 15, every 4 weeks; SP, S‐1 80 mg/m^2^/day on days 1‐21 and cisplatin 60 mg/m^2^ on day 8, every 5 weeks; S‐1 + PTX iv + PTX ip, S‐1 80 mg/m^2^/day on days 1‐14, intravenous PTX 50 mg/m^2^ on days 1 and 8, and intraperitoneal PTX 20 mg/m^2^ on days 1 and 8; Bolus 5‐FU, 600 mg/m^2^ on day 1, every week.

Abbreviations: 5‐FU, 5‐fluorouracil; ci, continuous infusion; ip, intraperitoneal administration; iv, intravenous administration; MTX, methotrexate; OS, overall survival; PTX, paclitaxel; retro, retrospective study.

Nivolumab monotherapy is administered for heavily treated gastric cancer according to the ATTRACTION‐2 study. However, in that study, the response rate was 11%, and only three patients had a complete response,^8^, ^13^ although patients with complete or partial response had long‐term response.^13^ Unfortunately, few patients responded, and hyperprogressive disease was common.^14^ Many studies have examined predictive factors of the response to ICIs to select patients who may respond to such treatment. Microsatellite instability is one such predictive factor. Janjigian et al reported that the efficacy of cytotoxic drugs was limited among patients with MSI‐H gastric cancer.^15^ According to the KEYNOTE‐158 study, which was a single‐arm phase II trial for MSI‐H solid tumors treated with pembrolizumab, the median OS of all patients was 27.8 months, and the median OS of MSI‐H gastric cancer patients was not reached.^16^ In the subanalysis of the KEYNOTE‐061 and −062 studies, which were phase III trials of second‐line and first‐line pembrolizumab, respectively, for gastric cancer patients, the median OS of patients with MSI‐H gastric cancer who received pembrolizumab was longer than that of those receiving chemotherapy.^12^, ^17^ In the current case, the patient had a durable response to third‐line nivolumab because she had MSI‐H gastric cancer. The first‐ and second‐line treatments were not effective, which is consistent with the previous report. The patient has been disease free for 2 years with nivolumab therapy. Therefore, we believe it is important to evaluate the microsatellite instability status before first‐line treatment, and if the patient has an MSI‐H tumor, the response should be evaluated early during first‐line treatment.

PD‐L1 and Epstein‐Barr virus infection have also been reported to be predictive factors for treatment response to ICIs.^18^ In the KEYNOTE‐062 study, 21 (11.5%) of 182 patients with a CPS ≥ 10 had MSI‐H tumors, whereas 33 (6.5%) of 506 patients with a CPS ≥ 1 had MSI‐H tumors.^17^ CPS is associated with MSI status, and gastric cancer patients with a CPS ≥ 10 have sustained response to ICIs.

Patients with pre‐existing autoimmune disease develop irAEs more frequently than patients without autoimmune disease.^19^, ^20^ In this case, our patient had rheumatoid arthritis but did not develop severe irAEs. However, patients should still be closely monitored for irAEs during and after ICI administration.

In conclusion, we report herein the first case of marked improvement of oral intake with third‐line nivolumab monotherapy for a gastric cancer patient with insufficient oral intake. This report highlights that ICIs should be administered for MSI‐H gastric cancer, regardless of the patient's general condition.

## CONFLICT OF INTEREST

None declared.

## AUTHOR CONTRIBUTIONS

TO: Collected information for the case and drafted the initial version of the manuscript. YN: Drafted the initial version of the manuscript. KM: Critical feedback and editing of manuscript. WH: Researched references. KM: Critically edited and revised the initial draft of the manuscript with regard to important intellectual content, with a focus on the psychiatric aspects. All authors discussed the case and commented on the manuscript at all stages and provided their final approval of the version to be published in Clinical Case Reports.
